# Motor unit discharge rate in dynamic movements of the aging soleus

**DOI:** 10.3389/fnhum.2014.00773

**Published:** 2014-09-29

**Authors:** Jouni Kallio, Karen Søgaard, Janne Avela, Paavo V. Komi, Harri Selänne, Vesa Linnamo

**Affiliations:** ^1^Department of Biology of Physical Activity, Neuromuscular Research Center, University of JyväskyläJyväskylä, Finland; ^2^Institute of Sports Science and Clinical Biomechanics, University of Southern DenmarkOdense, Denmark; ^3^LIKES, Foundation for Sport and Health SciencesJyväskylä, Finland; ^4^Vuokatti Snowpolis, Department of Biology of Physical Activity, Neuromuscular Research Center, University of JyväskyläJyväskylä, Finland

**Keywords:** aging, dynamic contraction, soleus, intramuscular electromyography, motor unit

## Abstract

Aging is related to a variety of changes at the muscular level. It seems that the age-related changes in motor unit activation are muscle- and intensity dependent. The purpose of this study was to examine the motor unit discharge rate (MUDR) in both isometric and dynamic contractions of the aging soleus muscle. Eight elderly males participated in the study. The subjects performed isometric and dynamic plantar flexions while seated in an ankle dynamometer. The force levels studied were 10, 20, 40, 60, 80 and 100% of the isometric (ISO) maximal voluntary contractions (MVC) in ISO and 10, 20 and 40% in concentric (CON) and eccentric (ECC) contractions. Soleus intramuscular EMG was recorded with bipolar fine-wire electrodes and decomposed to individual trains of motor unit discharges. In ISO the MUDR increased with each force level from 40 to 100% MVC. In dynamic contractions the descriptive analysis showed a higher MUDR in CON compared to ISO or ECC. The difficulties of recording single motor units in dynamic contractions, especially in the elderly is discussed.

## Introduction

It is well known that ageing is related to changes at the muscular level, leading to a decline in motor performance. However, it seems that such changes in motor unit activation are muscle- and intensity dependent. Several studies have found the elderly to have a lower discharge rate during high intensity contractions in muscles, like the first dorsal interosseous, tibialis anterior (Kamen et al., 1995; Klass et al., 2008) and vastus lateralis (Kamen and Knight, 2004). In contrast, the soleus motor unit discharge rate (MUDR) was found to be lower in the elderly only at low force-levels, and for other muscles no age related decrease in MUDR was found in studies by Howard et al. (1988) and Galganski et al. (1993).

So far age-related differences in motor unit discharge behavior have mainly been investigated in isometric contractions. A large number of studies in young subjects have shown that neuromuscular control of isometric and dynamic contractions differ in many ways, including MUDR (Tax et al., 1989; Howell et al., 1995; Søgaard et al., 1996, 1998; Kossev and Christova, 1998; Del Valle and Thomas, 2005; Pasquet et al., 2006; Altenburg et al., 2009; Kallio et al., 2013) and discharge patterns (Søgaard, 1995; Søgaard et al., 1998) and double discharges (Søgaard et al., 2001).

Considering how essential dynamic contractions are in daily life activities, it is important to investigate the MUDR patterns also in dynamic contractions. This paper extends the scope of our previous study from the effects of contraction type on MUDR in young males (Kallio et al., 2013) to the effects of aging.

## Materials and methods

Eight physically active old males (age: 69.1 ± 5.1 year height: 1.69 ± 0.4 m body mass: 75.3 ± 6.2 kg), volunteered as subjects for the study. Physical activity of the subjects was determined with a questionnaire, where the subjects wrote the type and frequency of their regular exercise routine. All men performed moderate to strenuous physical exercise three times a week or more. Before the measurements all of the subjects underwent a medical examination. Only subjects without any history of neuromuscular or vascular disease were approved for the study.

**Figure 1:**
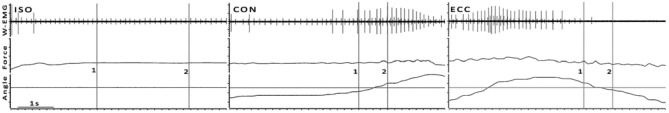
**Examples of ISO, CON and ECC trials from one subject at 10% MVC**. Cursors 1 and 2 mark the beginning and end of analysis, respectively. In dynamic contractions Cursor 1 is 200 ms prior and Cursor 2 200 ms after the angle crosses the angle of the ISO trial, as indicated by the horizontal cursor. This figure is a typical wire electrode recording (W-EMG), where the recruitment of an additional motor unit in CON and a derecruitment of active units in ECC can be observed (see Section Discussion for details).

The measurement protocol and analysis has been described in more detail in Kallio et al. (2013). Shortly, the subjects performed isometric and dynamic plantar flexions while seated in an ankle dynamometer. The test battery included (1) maximal voluntary contractions (MVC); (2) isometric contractions 10, 20, 40, 60, 80 and 100% of the isometric MVC; and (3) dynamic contractions 10, 20 and 40% of the isometric MVC: In the dynamic trials the subjects lifted concentric (CON) or lowered (ECC) a weight stack that was attached to the foot pedal via a cable pulley system at a voluntarily controlled velocity of 10°/s (Figure 1).

Global EMG activity of the soleus (SOL) and gastrocnemius medialis (GM) muscles was recorded using surface electrodes (Beckman 650437, USA), calculated as root mean square (RMS) of the signal and normalized relative to the maximal EMG at isometric MVC. For the intramuscular EMG recordings, four separate bipolar fine-wire electrodes were inserted into the soleus muscle. Signal decomposition, motor unit identification and data analysis were performed by utilizing the three channel decomposition technique computer algorithm, “Daisy” (Søgaard et al., 1996; Farina et al., 2001; Olsen et al., 2001). The MU classification was performed semi-automatically with a high degree of operator interaction.

The statistical analysis is limited to the isometric contractions, as the low number of subjects and analyzed units prevents statistical analysis for the dynamic contractions. The normality of distribution was tested with the Shapiro-Wilk test. In the data from the pooled motor units an analysis of variance (ANOVA) was used to compare the effects of contraction intensities. When a significant difference was detected, Bonferroni’s method was used to locate the difference. The critical level of significance was *P* < 0.05. Descriptive statistics include mean and standard deviation.

## Results

**Figure 2:**
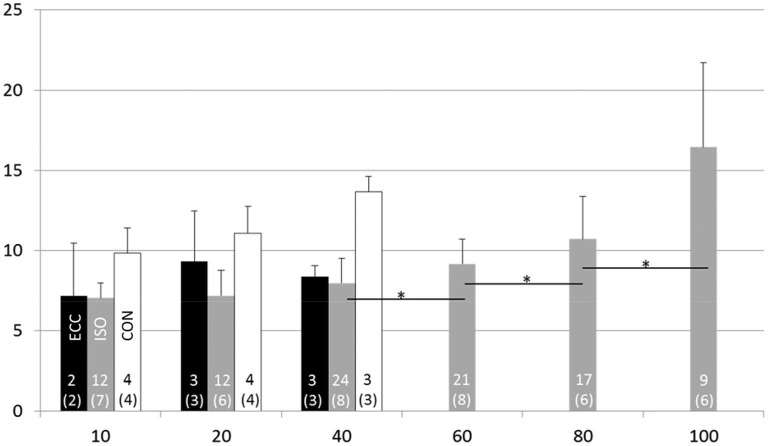
**Motor unit discharge rate (1/s; *X* ± SD) at different force levels in eccentric (ECC), isometric (ISO) and concentric (CON) contractions**. Number of motor units and subjects (in parenthesis) for each contraction type and activation level are displayed at the bottom of each bar. * = difference between action levels (*p* < 0.05).

The maximal voluntary isometric plantar flexion force was 149.2 ± 35.7 Nm. The MUDR analysis was based on a total of 2600 motor unit discharges 75 unique identified motor units. The number of subjects with decomposable units in each condition and the number of units can be seen in Figure 2. In ISO contractions, where force levels were explored up to 100% MVC, the MUDR increased significantly along with the increase in relative force level between 40 and 100% MVC (Figure 2). The increase followed a second order polynomial equation (*y* = 0.6*x*^2^ −2.2*x* + 9.0, *R*^2^ = 0.96). Similarly the relative sEMG activity of Soleus and Gastrocnemius increased with every step increase in force after 20% MVC. At the three lowest force levels all three contraction types were measured and according to the descriptive analysis the MUDR (Figure 2) and the RMS of surface EMG (Table 1) were higher in concentric (CON) compared to isometric (ISO) and ECC.

**Table 1:** **Relative surface EMG levels of soleus (SOL) and gastrocnemius medialis (GM) in different levels and contraction types, calculated relative to isometric MVC**.

**Type**	**Level(%)**	**SOL%**	**GM%**
ECC	10	31.8 ± 10.1	39.7 ± 0.2
	20	39.5 ± 16.7	34.0 ± 13.5
	40	43.3 ± 6.0	70.1 ± 20.1
ISO	10	20.9 ± 7.5	15.8 ± 4.1
	20	31.2 ± 6.7*	23.3 ± 7.2*
	40	47.5 ± 7.7*	32.8 ± 14.7*
	60	63.6 ± 6.9*	50.5 ± 16.0*
	80	73.3 ± 6.2*	81.8 ± 20.3*
	100	96.9 ± 10.9*	128.4 ± 32.2*
CON	10	47.2 ± 19.6	18.4 ± 2.1
	20	61.8 ± 20.7	42.2 ± 5.1
	40	70 ± 12.6	53.1 ± 20.5

* Significantly higher than in lower force level. Statistics calculated only for ISO, see text for details.

## Discussion

The main finding in the present study is that the in elderly males CON contractions required a higher MUDR than ECC or isometric contractions to reach the same relative force level.

As stated in the methods section, the same study has been conducted with adult males, aged 20–30 years (Kallio et al., 2013). In comparison, the current results show that in ISO trials MUDR was significantly lower in the elderly in most measured force levels (10, 40, 60 and 80% MVC). Similarly, both SOL and GA s-EMG-levels were significantly lower in the elderly (10, 20, 40 and 60% MVC). The observed age-difference in MUDR confirms the results of our previous measurements in isometric conditions (Kallio et al., 2012, 2013), as well as in submaximal dynamic (Kallio et al., 2010) contractions matched by relative surface EMG instead of force. Dalton et al. (2009) found in isometric condition an age difference in soleus MUDR at 25 and 50% MVC, but not at the higher contraction levels. Like the current study Dalton also reported a force-related increase in MUDR that was most pronounced at the high intensities in the elderly. The present results for the soleus muscle support the findings in other muscles, showing the age-difference in MUDR to be largest at high force levels (Kamen et al., 1995; Patten and Kamen, 2000; Kamen and Knight, 2004; Barry et al., 2007). Overall the current knowledge seems to suggest that the age-related changes in on MUDR are similar for the plantar flexor and the ankle dorsal flexor tibialis anterior (Connelly et al., 1999; Klass et al., 2008), as well as the small hand muscles first dorsal interosseous (Kamen et al., 1995) and adductor digiti minimi (Nelson et al., 1984). The decreased MUDR in the elderly has been suggested to be an adaptation to the increased twitch duration to optimize force generation (Roos et al., 1997). Previous studies have confirmed that the triceps surae twitch duration actually increases with age, suggesting that tetanus may be achieved with lower discharge rates (Dalton et al., 2009, 2010; Kallio et al., 2012).

Unfortunately the number of identifiable motor units was very low in the dynamic contractions. This was unexpected, considering that in the same study protocol with eleven younger subjects the number of units was between 2 and 9 times higher. Measuring and analyzing single motor units in dynamic contractions in elderly is very challenging, especially in ankle joint that has quite a small range of motion. Accelerating and controlling the ankle extension velocity is not easy, and the combination of a good MU recording and a kinematically successful trial was rare. The lower number of kinematically successful trials we were able to measure with the elderly explains only partially the lower N in current study. Why we had so few clear intramuscular EMG-recordings compared to the previous study is not clear. It could be speculated, that the increase in subcutaneous body fat could cause some of the wire-electrodes to have been placed too superficially in the elderly. Although we did not measure the body fat in our subjects, the small difference in BMI (25 in young, 26 in elderly) would not support this hypothesis.

It can be questioned if the age-related deterioration in muscle performance, like decreasing maximal force and slowing of MUDR and force production, is just an inevitable part of ageing or if it can be partly explained by an age related change in everyday activity. In the present study all the elderly subjects were as active as the younger subjects in our previous study (Kallio et al., 2013). Still, the isometric MVC was 29.3% lower (*p* < 0.01) compared to adult males. However, the open-ended questionnaire also showed clear differences in the types of activities young and elderly engaged in. The young subjects were more active in sports requiring strength and speed (e.g., running, soccer, ice-hockey, weight-training, tennis), than the elderly who mainly were walking with and without poles, swimming and gardening).

The descriptive analysis showed the largest MUDR in CON. Since we controlled the weights so that the absolute plantarflexion forces in all intensities were equal between contraction types, the most likely reason for the difference is found in the cross-bridge physiology. It has been shown that the force production of the cross-bridge is decreased when producing movement in CON contractions (Joyce et al., 1969). For this reason, to generate an equal amount of force, more activity either in terms of more recruited motor units or higher discharge frequency is required in CON compared to ISO or ECC contractions, which was also apparent as an increased surface EMG.

The joint rotation velocity (10°/s) in the current study was quite slow compared to most natural movements. For example, in walking, the angular velocity in dorsiflexion is ~30°/s and in plantarflexion ~220°/s (estimated from Lichtwark et al., 2007). The next challenge for motor unit studies would be to see how the activation functions in normal locomotion.

To our knowledge, this is the first attempt to record the effects of aging on the MUDR in dynamic contractions of large leg extensors. Based on our limited data the increase in MUDR when performing CON contractions was found to be similar to that of younger men. In isometric contractions the MUDR of the elderly men was lower compared to younger men in most measured force levels.

## Conflict of interest statement

The authors declare that the research was conducted in the absence of any commercial or financial relationships that could be construed as a potential conflict of interest.
